# Selective blockade of acid-sensing ion channel 1a can provide substantial hippocampal neuroprotection

**DOI:** 10.3389/fcell.2025.1582970

**Published:** 2025-07-03

**Authors:** Jiaai Li, Yu Cheng, Di Ma, Guangjian Li, Weixuan Zhao, Ting Jiang, Hongmei Meng

**Affiliations:** ^1^ Department of Neurology, The First Hospital of Jilin University, Changchun, China; ^2^ Department of Shanghai Mental Health Center, Shanghai Jiao Tong University School of Medicine, Shanghai, China

**Keywords:** ASIC1a, temporal lobe epilepsy, neuroprotection, hippocampus, Rho/ROCK, PI3K

## Abstract

**Background:**

Acid-sensing ion channel 1a (ASIC1a) is the only member of the ASIC family where Ca^2+^ osmosis has been reported, and it is highly expressed in neurons of the central nervous system. This study aimed to investigate whether ASIC1a is trafficked to the plasma membrane and regulated by the Rho/ROCK and PI3K signaling pathways in temporal lobe epilepsy (TLE). In addition, further research is required to determine whether selective ASIC1a blockade is a viable therapeutic strategy for TLE.

**Methods:**

The localization and expression levels of ASIC1 and mRNA levels of ASIC1a were detected when the Rho/ROCK and PI3K signaling pathways were activated and inhibited in glutamate (Glu)-induced cell. Meanwhile, we analyzed the location and expression of ASIC1 using Western blotting and immunofluorescence in brain tissue samples from TLE patients, kainic acid (KA)-treated rats, and Glu-induced primary hippocampal neurons. Currently, no specific ASIC1a antibody is available, so the ASIC1 antibody was used in this study, as in previous studies. Furthermore, we evaluated the HT22 cell survival rate, mitochondrial damage, apoptosis, and autophagy to examine whether selective blocking ASIC1a (PcTx1) could play a neuroprotective role.

**Results:**

First, the Rho/ROCK and PI3K signaling pathways affect the regulation of the expression and localization of ASIC1, especially the mRNA levels of ASIC1a in the Glu-induced HT22 cell injury model. Second, the high expression of ASIC1 in epilepsy patients was verified in all three sample types, and the phenomenon of its transport from the cytoplasm to the cell membrane/mitochondria was confirmed. Finally, although ASIC1 has a limited epileptogenic effect in the acute phase of epilepsy *in vivo*, selective blockade of ASIC1a using PcTx1 provided significant hippocampal neuroprotection and reduced mitochondrial damage, apoptosis, and cellular autophagy *in vitro*.

**Interpretation:**

This study is a systematic report concerning ASIC1a in temporal lobe epilepsy, including *in vivo* and *in vitro* experiments addressing both the acute and chronic phases. It provides foundational research for proposing ASIC1a as a new target for epilepsy treatment.

## Highlights

• The Rho/ROCK signaling pathway is involved in the regulation of the expression and localization of ASIC1, especially at the mRNA levels.
•ASIC1 is highly expressed in temporal lobe epilepsy accompanied by significant nerve fiber arrangement disorders.
•PcTx1, as a selective blockade of ASIC1a, can provide substantial hippocampal neuroprotection *in vitro*.

## Background

Epilepsy is one of the most common serious brain conditions, affecting over 70 million people worldwide (Thijs et al., 2019), and over a third of the patients develop drug-resistant epilepsy (Sultana et al., 2021). Temporal lobe epilepsy (TLE) is the most common type of refractory epilepsy.

Acid-sensing ion channels (ASICs) are proton receptors in the brain, belonging to the family of proton-gated cation channels. There are six subtypes (Boscardin et al., 2016). Acid-sensing ion channel 1a (ASIC1a) is the only member of the ASIC family where Ca^2+^ osmosis has been reported, and it is highly expressed in the neurons of the central nervous system. ASIC1a can mediate a large influx of Na^+^ and Ca^2+^ when cells are in an acidic environment (González-Inchauspe et al., 2017), which, in turn, enhances the opening of voltage-gated Na^+^ and voltage-gated Ca^2+^ channels, promotes the release of related neurotransmitters, and can even cause neuronal damage (Yermolaieva et al., 2004; Xiong et al., 2004). Blocking ASIC1a plays a neuroprotective role in various disease models, such as cerebral ischemia and Parkinson’s disease (Leng et al., 2016; Arias et al., 2008; McCarthy et al., 2015; Qiang et al., 2018).

With the deepening of research on the specific role of ASIC1a in epileptic diseases, scholars have found a significant association between the polymorphism of the ASIC1a gene and TLE (Lv et al., 2011). In a previous study, an ASIC1a variant allele (rs844347: A>C) was significantly associated with TLE, which suggested that ASIC1a may be a novel target for developing disease-modifying therapies in TLE (Lv et al., 2011). It has been demonstrated that increased Ca^2+^ inflow, mediated by ASIC1a, is closely related to ASIC1a plasma membrane transportation, especially the membrane/cytoplasm ratio of ASIC1a instead of an increased expression of ASIC1a protein (Herbert et al., 2018). In addition, this phenomenon is affected by the Rho/ROCK and PI3K signaling pathways. In a mouse model of epilepsy, research found that the membrane-to-plasma ratio of Rho was significantly higher than that of the control group (Liu et al., 2018), and treatment with ROCK inhibitors contributed to the survival of neurons in the epilepsy model (Ding et al., 2010; Inan and Büyükafşar, 2008; Gisselsson et al., 2010). Moreover, RhoA activation can increase ASIC1a-mediated Ca^2+^ influx by promoting ASIC1a transport to the cell membrane (Herbert et al., 2018), revealing the regulatory role of RhoA in the distribution and function of ASIC1a. The PI3K signaling pathway is involved in regulating a wide range of physiological and pathological activities, including cell growth, proliferation, metabolism, transcription, and protein synthesis (Zhang et al., 2018). PI3K belongs to a family of lipid kinases characterized by their ability to phosphorylate the inositol ring 3′-OH group in inositol phospholipids in the plasma membrane. PI3K and downstream effector Akt belong to a conserved family of signal transduction enzymes involved in the regulation of cell activation, inflammation, and apoptosis. Studies have indicated that the BDNF/TrkB pathway enhanced ASIC1a currents via phosphoinositide 3-kinase (PI3K)-protein kinase B (PI3K/Akt) cascade and phosphorylation of cytoplasmic residue Ser-25 of ASIC1a, resulting in enhanced ASIC1a trafficking to the neuronal surface and increased surface expression (Duan et al., 2012). In a mouse model of hepatic fibrosis, the PI3K/Akt pathway mediates the activation and migration of ASIC1a to the cell membrane, leading to calcium inflow and ER stress (Zuo et al., 2019). Although the correlation between ASIC1a and epilepsy, as well as its plasma membrane transport, has gradually gained researchers’ attention, no study to date has confirmed the regulation of ASIC1a by the Rho/ROCK and PI3K signaling pathways in epilepsy, which needs more experimental data.

This study is a systematic report concerning ASIC1a in temporal lobe epilepsy, including *in vivo* and *in vitro* experiments addressing both the acute and chronic phases. This study aimed to investigate whether ASIC1a is trafficked to the plasma membrane and regulated by the Rho/ROCK and PI3K signaling pathways in TLE, and further research is required to determine whether selective ASIC1a blockade is a viable therapeutic strategy for TLE.

## Materials and methods

### TLE and normal temporal cortex brain tissue

Control group: tissue samples were collected from the normal brain tissue of the focal edge of five glioma patients with no history of epilepsy in the Department of Neurosurgery, the First Hospital of Jilin University. The control inclusion criteria were as follows: a) after consultation and discussion with several neurosurgeons at the sub-senior level and above, it was confirmed that the normal temporal cortex tissue could not be avoided during brain tumor resection; b) no previous use of anti-seizure medicines (ASMs), no typical epileptic symptoms, and no family history of epilepsy; and c) the postoperative pathological examination results of the sample area in the study showed relatively normal brain tissue. Epilepsy group: the tissue samples were derived from the temporal cortex tissues of six TLE patients who underwent epileptogenic lesion resection in the Department of Neurosurgery of the First Hospital of Jilin University. Patients were enrolled according to the following criteria: a) typical epileptic symptoms and electroencephalogram (EEG) characteristics, with seizures lasting for more than 2 years; b) treatment with three or more ASMs without effective seizure control despite good tolerance; c) absence of other neurological diseases or underlying causes of symptoms unrelated to epilepsy; d) preoperative CT, MRI, and other head examinations showing no lesions; and e) diagnosis of primary temporal lobe epilepsy confirmed by several neurosurgeons at or above grade IV.

Fresh brain tissue was fixed in 4% paraformaldehyde overnight at 4°C. Following gradient alcohol dehydration, specimens were embedded in paraffin. Ethical approval for this study was obtained from the Ethics Committee of The First Hospital of Jilin University (approval no. 2022-142). Written informed consent was provided by all patients involved.

### Animals

A total of 179 adult male Wistar rats of specific pathogen-free (SPF) grade, with body weights ranging from 260–280 g, were used in this study. The animals were maintained under controlled environmental conditions at a temperature of 22°C–28°C and relative humidity of 50%–60%, with a 12-h light/dark cycle (with lights switched on at 8:00 a.m.). Food and water were provided to the animals *ad libitum*. Approval for all experimental procedures was obtained from the Animal Ethics Committee of the First Hospital of Jilin University (approval no. 2022-0182).

### KA TLE rats

The healthy rats were anesthetized with an intraperitoneal injection of 1 mL 10% chloral hydrate. The rats’ heads were fixed and disinfected. After disinfection and positioning (2.5 mm posterior to the fontanel and 4.5 mm lateral to the right side of the skull at the dural surface), the needle was slowly injected to a depth of 8.5 mm beneath the skull, and kainic acid (KA) (60 μL) was slowly injected. The same volume of PBS buffer was slowly injected, and the needle was left for 10 min after injection and then slowly withdrawn in the sham group. The Racine five-level epileptic behavior method was used to grade and evaluate seizures. Rats that experienced seizures of grade IV or above for 1 consecutive hour were selected for subsequent experiments and were euthanized by intraperitoneal injection of diazepam (8 mg/kg) after 1 h of seizures.

### Primary cortical neurons

Hippocampal tissue was isolated from E16–E18 pregnant rats’ fetal heads, and neuron cells were cultured for 7–10 days. The primary cortical neurons of fetal rats were extracted and cultured. Then, β3-Tubulin was used to label the dendrites, and DAPI was used to label the nuclei to calculate the purity of neurons. When the purity of neurons was more than 90%, subsequent experiments could be conducted.

### Cell culture, passage, and cryopreservation of HT22 cells

Frozen cells were thawed at 37°C, gently mixed with complete culture medium in a ratio of 1:3, and centrifuged at 4°C (800 rpm) for 5 min. The supernatant was discarded while retaining the cell pellet. Cells were resuspended in 5 mL of complete medium by gentle pipetting to ensure even distribution. The cell suspension was then transferred to a 25 cm^2^ ventilated culture flask and maintained under standard conditions (37°C and 5% CO_2_). Once cell confluence reached 80%–90%, passaging was performed using trypsin digestion. Microscopic observation confirmed the completion of digestion when cells displayed rounded morphology. Cells were gently triturated to break up clusters and subsequently centrifuged at 4°C (1,200 rpm). The resulting pellet was resuspended in 10 mL of complete medium, and a 1:4 dilution of the cell suspension was used for further culture. Cells that had been passaged three times following recovery were designated for cryopreservation.

We chose to use the HT22 cell line (FuHeng, FH1027, China) to construct a Glu cell-damage model based on the following factors: 1) KA is an analog of L-GLU, which can simulate the excitatory toxicity of Glu *in vivo* under pathological conditions; 2) neuronal death in epileptic pathology was mainly caused by Glu through both receptor and non-receptor pathways (Vishnoi et al., 2016); 3) the surface of the HT22 cell line of mouse hippocampal neurons does not express ionic Glu receptors, making it a good model to study the oxidative damage of Glu in the nervous system; 4) multiple studies have shown the functional interactions between ASIC1a and NMDAR in regulating hippocampal synaptic plasticity (Buta et al., 2015; Liu et al., 2016). By using HT22 cell lines without non-ionic Glu receptors, we can avoid such interactions interfering with experimental results.

### Glu-induced HT22 cells

Considering the results of detecting the cell viability by CCK-8 (Figures 5B, C) and the treatment conditions of other studies, we determined the following treatment conditions for this experiment: Glu concentration of 5 mM and treatment time of 6 h.

### Cell activity was detected by CCK-8

An amount of 100 μL containing 5,000 cells was added to each well in a 96-well plate. Following the incubation period, specific drug stimulations were applied to the cultures. Subsequently, 10 μL of enhanced CCK-8 solution was added to each well. The plate was then returned to the cell incubator for an additional 1 h of incubation. Afterward, absorbance at 450 nm was measured using a microplate reader (Bio-TEX, United States).

### Extraction of total protein

A 1 mM PMSF solution was prepared. The right hippocampus was extracted from each rat and subsequently weighed. The tissue sample was then minced into small fragments. A volume of 150–250 μL of lysate per 20 mg of tissue was added. Following the cracking procedure, the sample was centrifuged at 10,000–14,000 g for 3–5 min. The supernatant was collected.

### Extraction of mitochondrial protein

A total of 2 × 10^7^ cells were obtained by cell scraper, and 500 g of tissue was centrifuged for 5 min. Suspended cells were collected and precipitated (cold PBS). A total of 500 g was centrifuged for 2–3 min, and PBS was discarded. After the cell precipitates were collected and 0.75 mL lysis buffer A (1×, ExKine™ Mitochondrion Extraction Kit, KTP4004, United States) was added, the cells were swirled for 10 s at half the maximum speed. After incubation on ice for 2 min, the cell suspension was transferred to a homogenizer (on ice). The 250 µL lysis buffer C (1×, ExKine™ Mitochondrion Extraction Kit, KTP4004, United States) was added to the cell suspension and centrifuged in a test tube (600 g, 4°C, 10 min). The supernatant was collected and centrifuged (4°C, 3,000 g, 15 min). After removing the supernatant, the precipitate was suspended in 200 µL storage buffer and frozen at −80°C.

### Cell total membrane protein extraction

The cells were suspended in PBS and centrifuged (1,000 g, 5 min). After the supernatant was discarded, cytoplasmic protein release buffer (Abmart, A10008) was added (2 mL/1 × 10^7^ cells) and thoroughly mixed (shaking at 4°C, 10 min). After centrifugation (14,000 rpm, 4°C, 10 min), the supernatant cytoplasmic protein was collected, precipitated, added to buffer C (0.2 mL/1 × 10^7^), mixed (shaking at 4°C, 30 min), and finally centrifuged (14,000 rpm, 4°C, 10 min). The supernatant is the extract of the cell’s total membrane protein. The extracted total membrane proteins included the membranes of organelles such as mitochondria, endoplasmic reticulum, and Golgi apparatus.

### Mitochondrial fluorescence staining

To date, the only specific ASIC1a antibody is the one generated by Qiang et al. (2018), which is not commercially available. After contacting some authors who claimed to have used the ASIC1a antibody and confirming that they had actually used the total ASIC1 antibody, we chose the anti-ASIC1 primary antibody (ab240896, Abcam) for this study.

A small amount of 200 μM MitoTracker Red CMXRos storage solution was added to the cell culture solution (DMEM with 10% fetal bovine serum, same as below) to prepare a 100 nM working solution. After the cells were treated in confocal culture dishes, the culture medium was removed, and the working medium was added and incubated for 20 min (37°C). After the solution was removed, fresh cell culture solution was added (37°C) for staining. After washing the cells with fresh cell culture solution, the culture solution was removed, and the complete culture solution with fresh formaldehyde was added (4°C, 10 min). The medium was aspirated, and the cells were covered with pre-cooled 100% methanol. The samples were fixed on ice or at 4°C for 15 min. The sections were then washed three times with PBS, with each wash lasting 5 minutes. Following this, the specimens were blocked in a blocking buffer for 60 min. After blocking, the solution was aspirated, and a diluted anti-ASIC1 primary antibody (ab240896, Abcam) was added. The samples were then incubated at 4°C overnight. Subsequently, the sections were washed three times with PBS, with each wash lasting 5 minutes. Next, the fluorescence-conjugated goat anti-mouse IgG (H + L) cross-adsorbed secondary antibody (A-11001, Thermo Fisher Scientific) was diluted in antibody dilution buffer, and the samples were incubated in the dark for 1 hour. Afterward, the sections were washed three times with 1X PBS, each wash lasting 5 minutes, under dark conditions. Finally, DAPI staining for nuclei was applied, and the slides were mounted with an anti-fade mounting medium to prevent fluorescence quenching.

### Cell immunofluorescence staining

Brain tissue sections from both patient and control groups, as well as rat cortical tissue, were subjected to the following processes: first, recombinant anti-calponin 3 antibody (ab314329, Abcam) was applied at a dilution of 1:200 and incubated for over 12 h at 4°C. Following this step, the sections were removed and allowed to rewarm at room temperature for 1 h. The slides were then immersed in PBST for 3 min and washed five times. Subsequently, goat anti-rabbit IgG (H + L) cross-adsorbed secondary antibody (A-11008, Thermo Fisher Scientific) was administered and incubated at room temperature for 2 h. The slides were again immersed in PBST for 3 min and washed five times. To minimize nonspecific binding, the sections were blocked with glycine for a duration of 40 min. This was followed by three washes in PBST, with each lasting 5 min. Next, an anti-ASIC1 antibody (ab240896, Abcam) was applied and incubated overnight at 4°C. After removal from the refrigerator, the sections were allowed to rewarm at room temperature for 1 h. The slides were then immersed in PBST three times, with each immersion lasting 5 min. Goat anti-mouse IgG (H + L) cross-adsorbed secondary antibody (A-11005, Thermo Fisher Scientific) was subsequently administered and incubated at room temperature for 1 h. The slides were then washed three times in PBST, with each wash lasting 5 min. Finally, DAPI staining was performed to label nuclear DNA. Following this step, the slides were mounted with an anti-fluorescence quenching agent and stored in darkness at 4°C to prevent photobleaching.

Immunofluorescence staining of HT22 cells was conducted following the same methodology as described above. The primary antibodies used in this procedure included an anti-ASIC1 antibody (ab240896, Abcam) and a recombinant anti-Rho antibody [EP487Y] (ab40673, Abcam). The secondary antibodies employed were goat anti-mouse IgG (H + L) cross-adsorbed secondary antibody (Alexa Fluor™ 488, A-11001, Thermo Fisher Scientific) and goat anti-rabbit IgG (H + L) cross-adsorbed secondary antibody (Alexa Fluor™ 594, A-11012, Thermo Fisher Scientific).

### Western blotting

Protein samples were separated by 10% SDS–polyacrylamide gel and then transferred to polyvinyl difluoride membranes (Millipore, United States), which were then blocked for 1 h with 5% skim milk in TBST [10 mM Tris, 150 mM NaCl, and 0.05% Tween 20 (pH 8.3)] at room temperature. The membranes were incubated overnight at 4°C with anti-ASIC1 antibody (Abcam, ab284406, UK) or anti-Rho antibody (Abcam, ab40673, UK), anti-NFATc1 antibody (Abcam, ab177464, UK), anti-LC3B antibody (Abmart, T55992, China), anti-NFATc4 antibody (CST, 2188, United States), anti-Na+/K+-ATPase antibody (Abbkine, ABL1141, China), anti-COX antibody (Abbkine, ABL1060, China), anti-β-actin antibody (Bioss, bs-0061R, China), and anti-GAPDH antibody (Bioss, bs-10900R, China). The membranes were washed in TBST and incubated with a secondary antibody (1:10,000, Bioss, China) for 1 h at room temperature, followed by exposure to electrochemiluminescence. The results were expressed as a percentage of control signals in each blot to correct for variations between blots. The total membrane protein extraction kit was used in this study, and the extracted total membrane proteins included the membranes of organelles such as mitochondria, endoplasmic reticulum, and Golgi apparatus.

### Determination of mitochondrial membrane potentials

Five microliters of enhanced mitochondrial membrane potential assay kit with JC-1 (JC-1, Beyotime Biotechnology) at a 200X concentration were added to 1 mL of JC-1 staining buffer. The mixture was gently pipetted and repeatedly mixed. The culture medium was removed, and the cells were washed once with PBS. Subsequently, 1 mL of complete culture medium was added. One milliliter of JC-1 dyeing solution was then added, and the mixture was thoroughly blended. The samples were incubated at 37°C for 20 min. Following incubation, the supernatant was aspirated, and the cells were washed twice with JC-1 dyeing buffer. Two milliliters of complete cell culture medium were subsequently added, and the samples were examined under a fluorescence microscope (Olympus, Japan). Green fluorescence was observed to indicate a decrease in mitochondrial membrane potential, signifying early apoptosis. Red fluorescence was detected to represent normal mitochondrial membrane potential, reflecting cells in a healthy state.

### Annexin V-EGFP was used to detect apoptosis

The cells were cultured in confocal dishes coated with polylysine. After drug treatment, the cell culture medium was removed and the cells were gently washed with PBS. The supernatant was removed. Staining buffer was prepared by adding 4 µL Annexin V-EGFP and 4 µL propyl iodide (PI) into 192 µL binding buffer, followed by mixing and centrifugation. The staining buffer was applied to the cells, which were then incubated in the dark (at room temperature or 37°C for 5–10 min). After incubation, the staining buffer was removed, and the cells were immediately observed under a fluorescence microscope.

### Data statistics

All values are expressed as the mean ± SEM unless otherwise specified, and each experiment was repeated at least three times independently. Statistical analysis was performed using GraphPad Prism 8.0 software. Statistical comparisons were performed using Student’s *t* tests or one-way ANOVAs. In cases where a significant statistical difference was found, Tukey’s test was used for multiple comparisons. The statistical results indicated a significant difference when p < 0.05 and a highly significant difference when p < 0.01.

## Results

### The Rho/ROCK signaling pathway is involved in the regulation of ASIC1 expression and localization

#### The expression of ASIC1 and Rho changed synchronously in fluorescence colocalization *in vitro*

**FIGURE 1:**
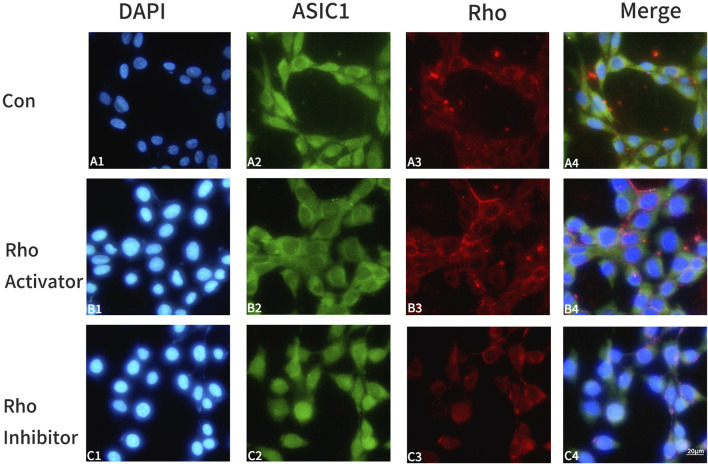
Changes in ASIC1 co‐locate with Rho fluorescence after the administration of Rho agonists/inhibitors in HT22 cells. Control group: A1‐A4; Rho activator group: B1‐B4; Rho inhibitor group: C1‐C4. DAPI (blue, cell nucleus), ASIC1 (green), and Rho (red) immunofluorescence staining was performed on the control group (Con, HT22 cells), Rho activator group (HT22 cells with 1 μg/mL Rho activator for 4 h), and Rho inhibitor group (HT22 cells with 1 μg/mL Rho inhibitor for 4 h).

HT22 cells were divided into three groups and treated with the same amount of DMEM medium, Rho activator (1 μg/mL), and Rho inhibitor (1 μg/mL) for 4 h. Rho and ASIC1 were labeled with double immunofluorescence staining. The results suggest that the activation of Rho enhanced the expression of ASIC1 in cells and promoted the transfer of ASIC1 from the perinuclear region to the cell membrane to a greater extent (Figures 1B2–B4); inhibition of Rho had the opposite effect (Figures 1C2–C4).

#### The cell membrane/cytoplasm ratio of Rho was similar to that of ASIC1 in Glu-induced HT22 cells

**FIGURE 2:**
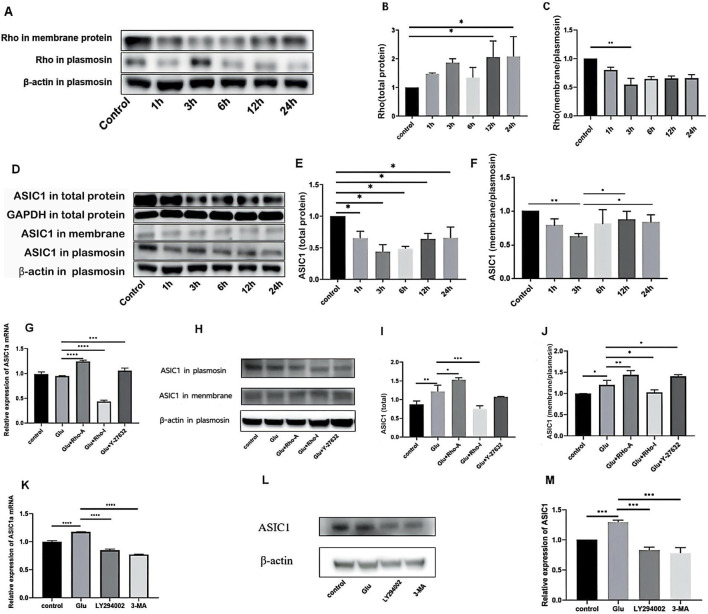
**(A)** Western blotting indicated that the expression levels of Rho in total protein and total membrane protein changed significantly over time after adding Glu. **(B)** Relative expression of Rho in total protein. **(C)** Ratio of the relative expression of Rho in total membrane proteins to relative expression in total proteins. **(D–F)** Changes in the expression levels of ASIC1 in total protein and total membrane protein fractions of primary cortical neurons after treatment with 5 mM Glu for different durations. The value of total protein minus total membrane protein was plasmosin protein. Statistical chart of the gray value: ASIC1 is normalized against β-actin and the optical density of the control group. **(G)** Changes in ASIC1a mRNA expression levels after the administration of different drug treatments (1 μg/mL Rho activator, 1 μg/mL Rho inhibitor, and 10 μM ROCK inhibitor Y-27632). **(H–J)** Changes in ASIC1 expression after treatment with different drugs (1 μg/mL Rho activator, 1 μg/mL Rho inhibitor, and 10 μM ROCK inhibitor Y-27632). **(K)** Changes in mRNA expression levels of cellular ASIC1a after treatment with different PI3K inhibition (15 μM LY294002 or 60 μM 3-MA). **(L,M)** Changes in ASIC1 protein expression levels after treatment with different PI3K inhibitors (15 μM LY294002 or 60 μM 3-MA). The optical density of each lane was represented by mean ± SD, and single-factor analysis of variance and the Tukey method were used for comparison between groups. *, p < 0.05; **, p < 0.01; ***, p < 0.001; and ****, p < 0.0001.

Rho in the total protein showed a trend of initially increasing, then decreasing, and then gradually increasing again (Figure 2A,B). The expression of ASIC1 in the total protein first decreased and then increased (Figure 2E). Similarly, the ratio of Rho in the cell membrane to cytoplasm first decreased and then increased gradually (Figure 2A,C), which was similar to the trend of ASIC1 (Figure 2F). It is reasonable to infer that in Glu-induced HT22 cells, ASIC1a is transported from the cytoplasm to the cell membrane, and this process may be regulated by Rho.

#### The Rho/ROCK signaling pathway affects ASIC1a expression and localization

Real-time PCR showed that the activation of Rho increased the expression of ASIC1a mRNA in cells, while the inhibition of Rho decreased the expression of ASIC1a mRNA. The inhibition of ROCK slightly increased the expression of ASIC1a mRNA (Figure 2G). Western blot analysis revealed that the expression of ASIC1 protein increased after Rho agonist administration, and the ratio of ASIC1 expression in the cell membrane to that in the cytoplasm increased. These results were reversed when Rho inhibitors were administered. After the administration of the ROCK inhibitor Y-27632, the expression of the ASIC1 protein did not change significantly, but the ratio of ASIC1 expression in the cell membrane was slightly increased compared to that in the cytoplasm (Figures 2H–J).

#### The inhibition of the PI3K signaling pathway can decrease ASIC1a expression

In Glu-induced HT22 cells, 15 μM LY294002 (a broad-spectrum PI3K inhibitor that inhibits PI3Kα, PI3Kδ, and PI3Kβ) and 60 μM 3-methyladenine (3-MA, a selective PI3K inhibitor that acts on Vps34 and PI3Kγ) were added before extracting cell mRNA, plasma proteins, and total membrane proteins. After the administration of LY294002 or 3-MA, expression decreased at both ASIC1a mRNA and ASIC1 protein levels (Figures 2K–M).

### ASIC1 is highly expressed in TLE and exhibits the transport phenomenon

#### ASIC1 showed increased expression and perinuclear aggregation in TLE patients

Calponin 3, also called acidic calponin, is one of the three isoforms of the calponin family that functions as calmodulin- and actin-binding partners, and calponin 3 is expressed in the central nervous system (Rami et al., 2006; Rozenblum et al., 2008). Calponin 3 is used in this study for nerve fiber localization.

**FIGURE 3:**
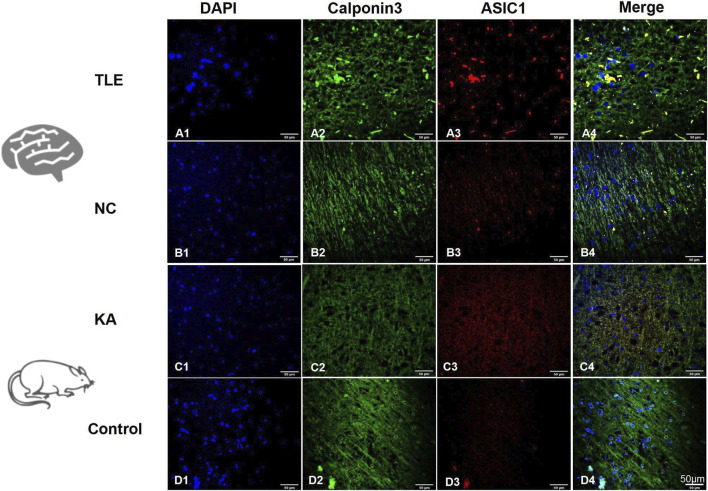
Expression and localization of ASIC1 in temporal lobe tissue of TLE patients and KA-treated rats. DAPI (blue, cell nucleus), calponin 3 (green, nerve fiber), and ASIC1 (red) immunofluorescence staining was performed on normal temporal lobe brain tissue (NC, normal control group: normal temporal cortex tissue removed incidentally during surgery in non-epileptic patients) and surgical resection specimens from temporal lobe cortex tissue of TLE patients.

The TLE group showed a significantly higher prevalence of nerve fiber disorder and abnormal arrangement than the normal control group (NC) based on calponin 3 immunofluorescence staining (Figures 3A2,B2). Immunofluorescence co-localization imaging revealed a more pronounced increase in ASIC1 expression (Figures 3A3,B3) and perinuclear aggregation (Figures 3A4, B4) in the TLE group, suggesting a close association between ASIC1 and abnormal nerve fiber development in the brain tissue of TLE patients.

#### ASIC1 was highly expressed and had changed localization in KA rats

**FIGURE 4:**
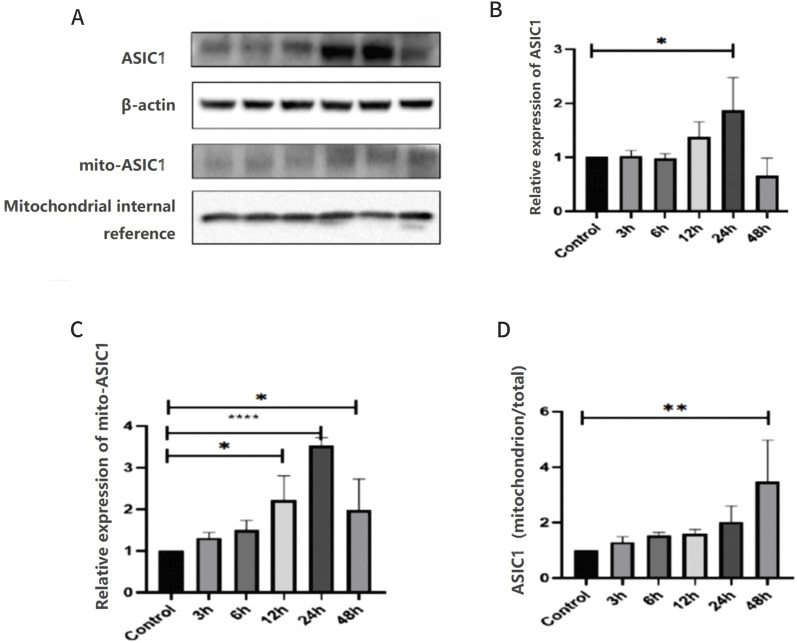
Expression and localization of ASIC1 were dynamically changed in KA-treated rats and Glu-induced primary hippocampal neurons. **(A)** Changes in the expression levels of ASIC1a in total protein and mitochondrial protein of hippocampal tissue at different time points after epileptic seizures. **(B)** Relative expression of ASIC1 at different time points. **(C)** Relative expression of mito-ASIC1 at different time points. **(D)** Ratio of ASIC1 expression in mitochondrial protein to total protein at different time points. The optical density of each lane was represented by mean ± SD, and single-factor analysis of variance and the Tukey method were used for comparison between groups. *, p < 0.05; **, p < 0.01; ***, p < 0.001; and ****, p < 0.0001. mito-ASIC1, ASIC1 in mitochondrial protein.

KA-treated rats and the control (sham operation) group were used, and the expression levels of ASIC1 in the total protein and mitochondrial protein of the hippocampus were examined at different time points after modeling. The expression of ASIC1 initially increased and then decreased, reaching its peak at 24 h (Figures 4A–C). The ratio of ASIC1 expression in mitochondrial protein to the total protein increased gradually over 48 h (Figure 4D).

The brain tissue slices of the temporal cortex of the two groups of rats were selected for immunofluorescence imaging after 24 h of modeling. At this time, the expression of ASIC1 was at its highest. In the control group, calponin 3 was uniformly distributed and arranged along the nerve fibers (Figure 3D2), and ASIC1 expression was almost at the level of non-expression or very low expression (Figure 3D3). Meanwhile, in the KA group, calponin 3 and ASIC1 were disordered along the nerve fibers, and ASIC1 expression increased significantly. ASIC1 and calponin 3 had almost the same distribution in the KA group (Figures 3C2–C4). These findings are consistent with the abnormal expression and distribution changes in ASIC1 in the temporal lobe brain tissue of TLE patients (Figures 3A2–A4).

### Selective blockade of ASIC1a can provide substantial hippocampal neuroprotection

#### PcTx1 reduces the incidence and severity of seizures

**FIGURE 5:**
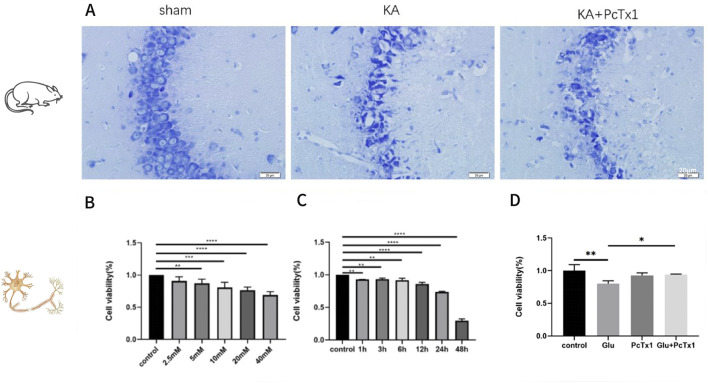
Role of PcTx1 in kainic acid-treated rats and Glu-induced primary hippocampal neurons. **(A)** Nissl staining in the CA3 region of hippocampus (400×): the neurons in the sham group were arranged neatly and had a normal morphology; the neurons in the KA group, CA 3 area, died and were clearly lost, and the remaining neurons were disorderly distributed. Neuronal loss in the PcTx1 group was not significantly different from that in the KA group. **(B)** CCK-8 was used to evaluate the cell viability of HT22 under different Glu concentrations. **(C)** Cell viability of HT22 treated with 5 mM Glu was evaluated by CCK-8 at different time points. **(D)** Effect of PcTx1 (2 nM) on HT22 cell survival (Glu: 5 mM; 6 h). (B–D)*, p < 0.05; **, p < 0.01; ***, p < 0.001; and ****, p <0.0001. Data represent the absorbance calculated using the corresponding formula and are expressed as the mean ± SD.

There were no epileptic seizures in the sham-operated group, while 91.67% of the rats in the KA group had seizures of grade IV or above. In the PcTx1 group, 75% of the rats had seizures of grade IV or above, but the degree of the seizures was lower on average. There was no significant difference in the condition of seizures and neuron death and loss in the CA3 area (Figure 5A) between the KA and PcTx1 groups.

#### PcTx1 improves the survival rate of Glu-induced HT22 cells

Glu-induced HT22 cells were established, and the experimental conditions (Figures 5B, C) were explored to determine the treatment conditions: Glu concentration of 5 mM and treatment time of 6 h. The results showed that the cell viability of the Glu group was significantly decreased than that of the control group, while PcTx1 increased the survival rate of the Glu injury cell model (Figure 5D).

#### PcTx1 ameliorated mitochondrial damage caused by Glu and partially restored the distribution of ASIC1 to normal

**FIGURE 6:**
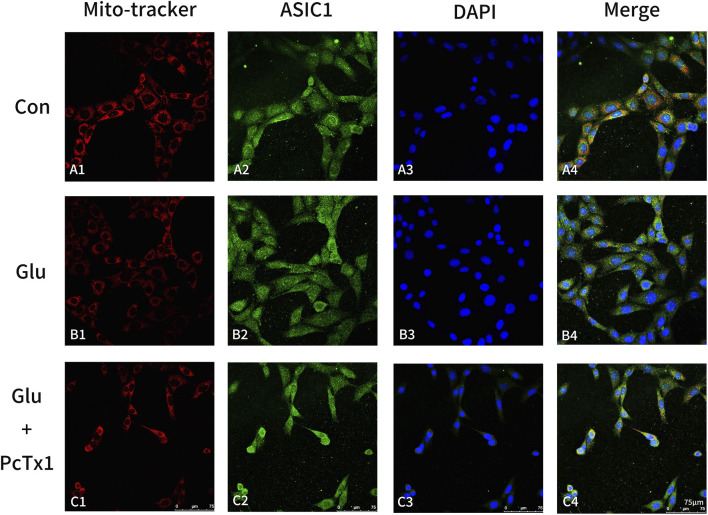
Blocking ASIC1a can alleviate mitochondrial damage. MitoTracker (red, mitochondria), ASIC1 (green), and DAPI (blue) immunofluorescence staining were performed on the control group (Con, HT22 cells: **(A1-A4)**), Glu group (Glu-induced HT22 cells: **(B1-B4)**), and Glu + PcTx1 group (Glu-induced HT22 cells with PcTx1: **(C1-C4)**). HT22 cells in each group were treated with the same amount of DMEM medium, 5 mM Glu, and 5 mM Glu + 2 nM PcTx1 for 6 h.

MitoTracker Red CMXRos was used to label the mitochondria of living cells, and then immunofluorescence staining of ASIC1 was performed. The results showed that in the control group, ASIC1 was mainly distributed on the perinuclear and mitochondrial membranes (Figures 6A1–A4). The fluorescent signal intensity of mitochondria decreased, while ASIC1 expression increased in the Glu-induced HT22 cell group (Figures 6B1, B2), and the distribution phenomena changed (Figure 6B4). The perinuclear aggregation of ASIC1 and its orderly distribution on the mitochondria were disrupted. Selective blocking of ASIC1a with PcTx1 ameliorated mitochondrial damage caused by Glu (Figure 6C1) and partially restored the distribution of ASIC1 to normal (Figure 6C4).

#### PcTx1 significantly reduces apoptosis and autophagy *in vitro*

**FIGURE 7:**
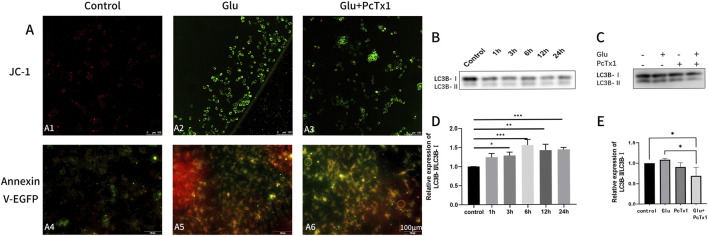
PcTx1 decreased mitochondrial apoptosis and autophagy of HT22 cells induced by Glu. **(A)** JC-1 staining group **(A1-A3)**: the transformation of JC-1 from red fluorescence (polymer) to green fluorescence (monomer) can be used as an early detection index of apoptosis. The Annexin V-EGFP staining group **(A4-A6)** revealed green fluorescence in apoptotic cells. Dead cells exhibit red and green fluorescence, while living cells show almost no fluorescence. Blocking ASIC1a was found to attenuate Glu‐induced apoptosis in both sets of staining. **(B,D)** Western blotting results showed that the ratio of LC3BⅡ to LC3BⅠ in HT22 cells changed after treatment with 5 mM Glu at different times. **(C, E)** Western blotting results showed that blocking ASIC1a attenuated the Glu‐induced elevation of the ratio of LC3BⅡ to LC3BⅠ in HT22 cells. *, p < 0.05; **, p < 0.01; ***, p < 0.001; and ****, p < 0.0001. Data represent the absorbance calculated using the corresponding formula and are expressed as the mean ± SD.

The mitochondrial membrane potential JC-1 kit and Annexin V-EGFP apoptosis kit were used to observe cell apoptosis. PcTx1 reduced cell apoptosis and autophagy caused by Glu (Figure 7A). The total protein amount and the expression of autophagy-related protein LC3-B in HT22 cells were detected in order to clarify the role of ASIC1 in Glu-induced autophagy. Autophagy initially increased and then decreased (the peak of autophagy occurred after 6 h) (Figure 7B,D). Compared with the control group, the autophagy of the Glu group was increased. PcTx1 significantly reduced the autophagy of HT22 cells induced by Glu (Figure 7C,E).

## Discussion

This study began by confirming that the Rho/ROCK and PI3K signaling pathways play a role in the regulation of ASIC1 expression and localization. It was found that a Rho activator can significantly promote ASIC1 plasma membrane transport. Second, we examined the expression and localization of ASIC1 in three distinct epilepsy models, which indicated that the upregulated expression of ASIC1 in TLE pathogenesis was accompanied by significant nerve fiber arrangement disorders and the phenomenon of cytoplasmic-to-cell membrane/mitochondria transport. Finally, it was verified that blocking ASIC1a can reduce KA/Glu-induced neuron damage and exert a neuroprotective effect at the systemic and cellular levels. This study is a systematic report concerning ASIC1 in temporal lobe epilepsy, including *in vivo* and *in vitro* experiments addressing both the acute and chronic phases. It provides foundational research evidence for proposing ASIC1a as a new target for epilepsy treatment.

### Role of ASIC1a in seizure

In previous studies, the exact role of ASIC1 in seizure onset, transmission, and termination remains controversial. On one hand, some studies have demonstrated that ASIC1a exerts a strong epileptogenic effect. For example, mice overexpressing ASIC1a showed higher sensitivity to chemically induced seizures, the expression level of ASIC1a was significantly increased in reactive astrocytes in the hippocampus of epileptic mice, and activated ASIC1a could significantly increase the concentration of calcium ions in reactive astrocytes. Additionally, knocking down or knocking out ASIC1a expression can reduce spontaneous seizures (Yang et al., 2016). Amiloride, a nonspecific blocker of ASIC1a, effectively inhibits epileptogenesis in a pilocarpine-induced epileptic rat model (Liang et al., 2015), and it can protect mice from a pentylenetetrazole-induced epileptic state by increasing the seizure threshold (Luszczki, 2009; Ali et al., 2004; Ali et al., 2006), delaying the latency of the first seizure in pilocarpine-induced epileptic rats, and reducing the occurrence of the epileptic state (N'Gouemo, 2008). However, amiloride can act on multiple ion channels (sodium hydrogen channel protein, epithelial sodium channel 17, etc.) (Velísek et al., 1994) and cannot exclude the interference of other channels. On the other hand, some studies suggest that ASIC1a also plays a role in the termination of seizures. ASIC1a^−/−^ mice experienced increased seizure duration. Similarly, the overexpression of ASIC1a in mice can suppress seizures (Ziemann et al., 2008). The different results of studies on the role of ASIC1a in epilepsy may relate to the different types of epilepsy studied, various animal models of epilepsy, and the non-specific ASIC1a blocker amiloride used in some studies, which also acts on other ion channels at the same time. Thus, this study focused on the TLE epilepsy type; selected the brain tissue from TLE patients, the KA epilepsy model, and the Glu-induced cell model, which are closer to the pathophysiological state of TLE; and used a specific ASIC1a blocker (PcTx1) to avoid interference.

### The function of ASIC1a is affected by its location and distribution

In addition to the expression of ASIC1a, its location and distribution have also gained scholarly attention. Previously, it was believed that ASIC1a was mainly expressed in the cell membrane and cytoplasm of neurons (Huang et al., 2010). However, a study in 2013 showed that the ASIC1a protein is also present in the mitochondria of mouse cortical neurons and may be an important regulator of mitochondrial permeability transition pores (Wang et al., 2013). Mitochondria are the primary site of cellular function (Liang et al., 2025). Furthermore, several studies have indicated that mtASIC1a plays an important role in acid-induced neuronal damage. In 2014, Li et al. demonstrated that extracellular acidosis could induce cytochrome C transport from mitochondria to the cytoplasm, leading to the apoptosis of endplate chondrocytes. This process can be inhibited by ASIC1A-siRNA or PcTX1 (Li et al., 2014). In chronic pain models, blocking the transport of ASIC1a to the membrane reduces pain sensitivity (Price et al., 2014), suggesting that the function of ASIC1a is closely related to its dynamic transport. This study also measured the localization of ASIC1 and the change in the plasma membrane ratio. It suggests that ASIC1 plays a central role in the occurrence and development of epilepsy, and plasma membrane transport may be crucial in this process.

### Regulation of ASIC1a function

Many factors are involved in the regulation of ASIC function (Velísek et al., 1994), including hydrogen ions, some aprotic ligands, protein kinases, and intracellular signaling molecules. Multiple protein kinases are involved in regulating the expression, function, and transport of ASIC1a (Ziemann et al., 2008; Huang et al., 2010; Wang et al., 2013). In this project, we chose to study the Rho/ROCK and PI3K signaling pathways based on the literature. Our study confirmed that the Rho/ROCK signaling pathway is involved in the regulation of ASIC1a expression and localization in the Glu injury model. Additionally, the Rho activator significantly promoted ASIC1a expression and ASIC1 plasma membrane transport. In addition, the expression of ASIC1a mRNA decreased along with ASIC1 protein levels after the administration of LY294002, a selective inhibitor of PI3Kγ, or 3-MA, a broad-spectrum inhibitor of PI3K. These results suggest the regulatory ability of the Rho/ROCK and PI3K pathways to regulate the expression and localization of ASIC1a.

### PcTx1 exerts neuroprotective effects in cell models of epilepsy

Seizures can cause the death of neurons in different forms. The dead neurons form a new abnormal network with the remaining neurons and new glial cells in the brain tissue. This network then becomes the cause of new epilepsy episodes, resulting in a cycle of repeated seizures. To better understand the function of ASIC1a in the pathology of neuronal injury in epilepsy, we evaluated its association with neuronal death both *in vitro* and *in vivo*. Selective blockade of ASIC1a (PcTx1) had a limited epileptogenic effect *in vivo* but increased the survival rate of Glu-induced HT22 cells and reduced the mitochondrial damage, apoptosis, and autophagy *in vitro* during the acute phase of epilepsy. This phenomenon may be attributed to limitations in the blood–brain barrier (BBB) permeability and potential compensatory mechanisms. PcTx1, a peptide toxin composed of 40 amino acids with a molecular mass of approximately 4.5 kDa (Escoubas et al., 2000), exhibits limited capacity for passive diffusion across the intact BBB. Furthermore, no experimental evidence has identified specific transport receptors of PcTx1. ASIC1a is present in mitochondria and may act as an important regulator of mitochondrial permeability transition pores (Wang et al., 2013). The protective effect of PcTx1 on mitochondria has been validated in other studies (Qiang et al., 2018; Li et al., 2014; Savic Azoulay et al., 2020). Apoptosis, a type of cell death, is known to play a key role in the occurrence and development of temporal lobe epilepsy. Simultaneously, the activation of the neuronal autophagy pathway is the main cause of neuronal loss in epilepsy models (Shao and Chen, 2017; Keezer et al., 2016; Yuen et al., 2018; Devinsky et al., 2016). The apoptotic and autophagy pathways have some common signaling molecular components, and the two processes interact to jointly regulate the degradation of intracellular proteins and organelles. It is suggested that the interaction between autophagy and apoptosis may play an important part in the loss of neurons in the epilepsy model (Tanaka et al., 1997). Our study confirms that PcTx1 can effectively reduce neuronal damage caused by Glu by inhibiting mitochondrial damage, autophagy, and apoptosis. PcTx1 exerts neuroprotective effects in cell models of epilepsy, which is consistent with the results of a 2024 study (Shi et al., 2024). This provides a strong theoretical basis for ASIC1a to be a new target for TLE treatment and neuronal protection.

The significance of this study is as follows: in terms of experimental methods, this study provides evidence that ASIC1a is involved in mediating neuronal damage in KA-treated rats and Glu-induced cells. We also verified that ASIC1 is trafficked to the plasma membrane and regulated by the Rho/ROCK and PI3K signaling pathways in temporal lobe epilepsy. PcTx1 ameliorated mitochondrial damage caused by Glu and partially restored the distribution of ASIC1 to normal, which supports a potential value of ASIC1a as a therapy target for TLE.

### Limitation

Although the present study has yielded some important findings concerning ASIC1a and its role in epileptogenesis, some limitations should be noted. At the cellular level, mitochondrial and membrane proteins could not be extracted due to technical reasons. This experiment could not detect the functional changes in ASIC1a or conduct specific experiments on acid-sensitive current changes. In the future, it is hoped that a new type of targeted nanoprobe can be used to label ASIC1a for *in vivo* imaging, providing more reliable results to support this study.

## Conclusion

1. The Rho/ROCK signaling pathway is involved in the regulation of ASIC1a expression and localization. The Rho activator can significantly promote ASIC1a plasma membrane transport.
2. ASIC1a is highly expressed in temporal lobe epilepsy accompanied by significant nerve fiber arrangement disorders and exhibits the transport phenomenon.
3. PcTx1 as a selective blockade of ASIC1a can provide substantial hippocampal neuroprotection.

## Data Availability

The raw data supporting the conclusions of this article will be made available by the authors, without undue reservation.

## Abbreviations

ASIC1a, acid-sensing ion channel 1a; TLE, temporal lobe epilepsy; ASMs, anti-seizure medicines; EEG, electroencephalogram; CT, computerized tomography; MRI, magnetic resonance imaging; SPF, specific pathogen free; KA, kainic acid.
